# Adaptive GDDA-BLAST: Fast and Efficient Algorithm for Protein Sequence Embedding

**DOI:** 10.1371/journal.pone.0013596

**Published:** 2010-10-22

**Authors:** Yoojin Hong, Jaewoo Kang, Dongwon Lee, Damian B. van Rossum

**Affiliations:** 1 Department of Computer Science and Engineering, The Pennsylvania State University, University Park, Pennsylvania, United States of America; 2 Center for Computational Proteomics, The Pennsylvania State University, University Park, Pennsylvania, United States of America; 3 Department of Computer Science and Engineering, Korea University, Seoul, Korea; 4 Department of Biostatistics, College of Medicine, Korea University, Seoul, Korea; 5 College of Information Sciences and Technology, The Pennsylvania State University, University Park, Pennsylvania, United States of America; 6 Department of Biology, The Pennsylvania State University, University Park, Pennsylvania, United States of America; BC Centre for Excellence in HIV/AIDS, Canada

## Abstract

A major computational challenge in the genomic era is annotating structure/function to the vast quantities of sequence information that is now available. This problem is illustrated by the fact that most proteins lack comprehensive annotations, even when experimental evidence exists. We previously theorized that embedded-alignment profiles (simply “alignment profiles” hereafter) provide a quantitative method that is capable of relating the structural and functional properties of proteins, as well as their evolutionary relationships. A key feature of alignment profiles lies in the interoperability of data format (e.g., alignment information, physio-chemical information, genomic information, etc.). Indeed, we have demonstrated that the Position Specific Scoring Matrices (PSSMs) are an informative M-dimension that is scored by quantitatively measuring the embedded or unmodified sequence alignments. Moreover, the information obtained from these alignments is informative, and remains so even in the “twilight zone” of sequence similarity (<25% identity) [1]–[5]. Although our previous embedding strategy was powerful, it suffered from contaminating alignments (embedded AND unmodified) and high computational costs. Herein, we describe the logic and algorithmic process for a heuristic embedding strategy named “Adaptive GDDA-BLAST.” Adaptive GDDA-BLAST is, on average, up to 19 times faster than, but has similar sensitivity to our previous method. Further, data are provided to demonstrate the benefits of embedded-alignment measurements in terms of detecting structural homology in highly divergent protein sequences and isolating secondary structural elements of transmembrane and ankyrin-repeat domains. Together, these advances allow further exploration of the embedded alignment data space within sufficiently large data sets to eventually induce relevant statistical inferences. We show that sequence embedding could serve as one of the vehicles for measurement of low-identity alignments and for incorporation thereof into high-performance PSSM-based alignment profiles.

## Introduction

One of the major challenges faced by biologists comes to this question: how to identify the relation between highly divergent protein sequences. Although numerous methods (e.g., [2], [6], [7]) have been proposed to address the problem, it still needs to be resolved. In general, when pairwise sequence identity between protein sequences fall below the 25% identity, statistical measurements fail to clearly identify phylogenetic relations, structural features or protein functions [2], [8]–[11]. GDDA-BLAST (Gestalt Domain Detection Algorithm - Basic Local Alignment Search Tool), originally introduced in [12], was designed to address the challenges arising in connection with low-identity alignments/divergence. It has been determined that this alignment information is informative to our laboratory experiments at multiple scales (e.g., whole protein, single protein domain and single amino acid) [3]–[5], [13]–[16]. The following analyses were put to use: (1) to reconstruct evolutionary histories, (2) to identify functions in the domains of the unknown function, (3) to classify structural homologues of high sequence divergence and (4) to isolate key amino acids important to protein function.

**Figure 1:**
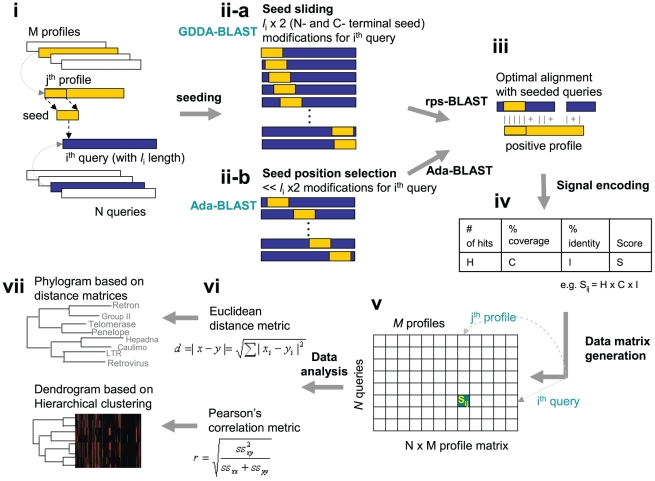
The Concept of GDDA-BLAST and Adaptive GDDA-BLAST. This schematic depicts the work flow of GDDA-BLAST and Adaptive GDDA-BLAST (i–ii) The algorithm begins with a modification of the query amino acid sequence via the insertion of a “seed” sequence from the profile of interest. These seeds are obtained from the profile consensus sequences from NCBI's Conserved Domain Database (CDD). GDDA-BLAST inserts a seed at every query amino acid position; in constrast, Adaptive GDDA-BLAST inserts a seed at the positions where the seed is likely to be extended to an alignment. (iii–iv) The signals are collected from the optimal alignments between the “embedded” sequences and profiles using rps-BLAST or Adaptive GDDA-BLAST; and, they are incorporated as a composite score into an *N* by *M* data matrix. (v–ix) This dataspace can be analyzed to generate phylograms and dendrograms based on the Euclidean distance and Pearson correlation measures on alignment profiles of query proteins, respectively.

A phylogenetic profile represents a protein as a vector where each entry quantifies the existence of the protein in a different genomes [17]–[19]. This approach has been proven applicable to whole molecules (Single Profile Method), to isolated domains (Multiple Profile Method) and to individual amino acids. Similar to phylogenetic profiles, our *embedded-alignment profiles* present a protein as a vector where each entry quantifies the existence of alignments with a PSSM [1], [2]. The basic idea underlying our method begins by compiling a set of PSSMs that the query sequence is compared to. These profiles are obtainable from any protein-sequence knowledge-base source (e.g., Protein Data Bank, Pfam, SMART, and NCBI Conserved Domain Database (CDD)) [20]–[23], or they are locally creatible through PSI-BLAST [24]. We employ the reverse specific position BLAST (rps-BLAST) [23] to compare the query with PSSMs. A single domain PSSM database is utilized for pairwise comparison. We then record and quantify all alignments between an unmodified (control) query sequence and a modified one. The latter is composed of two types of alignments: “seeded” and “non-seeded” alignments. We modify the query sequence with a “seed” from the PSSM, and, thereby, create a consistent initiation site (Figure 1-i, ii). The “seeds” are generated from the profiles by taking a portion (e.g., 10% in this study) of the PSSM sequence (e.g., from the N-terminus or C-terminus). These thresholds have been adopted herein from the results of our previous studies. These “seeds” are embedded at each position of the query sequence. For example, a query sequence of 100 amino acids yields 100 distinct test sequences for each “seed”. This strategy is designed to amplify and encode all the alignments possible for any given query sequence. In place of a sliding window, we utilized a sliding “seed,” which is similar, yet inverse to the embedding strategies employed by Henikoff and Henikoff [25].

Then, each of these modified query sequences is aligned by rps-BLAST against the parent profile, from which a seed is taken. Due to the “hit and the extension of the hit” approach of BLAST algorithm, the embedded “seed” creates a consistent initiation site that allows rps-BLAST to extend an alignment even between highly divergent sequences. The idea is summarily depicted in Figure 1-iii. Next, the alignments from rps-BLAST are filtered out, based on the thresholds of percentage identity and percentage coverage (i.e., the alignment length as a function of the profile length) in order to eliminate random alignments (Figure 1-iv). The output of the comparison is translated into a composite score of either zero (when there is no significant match) or a positive value (that measures the degree of successful match of the protein sequence to each of the profiles). The composite score is computed as a product of the number of hits, aveage pairwise identity of the hits, and maximum coverage of the hits. This procedure is readily adaptible to make an unbiased comparison between a series of query sequences by subjecting them to the same screening analysis with the same set of PSSM sequences. After the analysis, each query sequence (N) is represented in a vector of non-negative numbers in M dimensions (M = # of “PSSMs” tested) (Figure 1-v). This 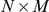 data matrix (N alignment profiles of length M for N queries) is then usable to create a dendrogram through distance metrics such as Euclidian distance or Pearson's correlation between query sequences (Figure 1-vi∼vii).

Despite the great potential of the embedded alignment strategies in answering a diverse set of biological questions, their computational costs are prohibitively expensive, a problem that arises out of the current method of generating and analyzing embedded sequences. As proteins range in length from tens of amino acids to <8000, the proteomic scale studies using GDDA-BLAST are prohibitive in nature. To address this challenge, in this paper, we propose a novel sequence alignment algorithm that is as sensitive as GDDA-BLAST but is orders of magnitude faster. *Ada*ptive *GDDA-BLAST* is termed for its adaptive nature, and exploits similarities among embedded sequences to adaptively avoid expensive computations. Instead of inserting a seed into every position of a query sequence, Adaptive GDDA-BLAST embeds a seed at the query positions where the seed is likely to be extended to an alignment (see the Method section for details).

## Results

### Execution time

**Figure 2:**
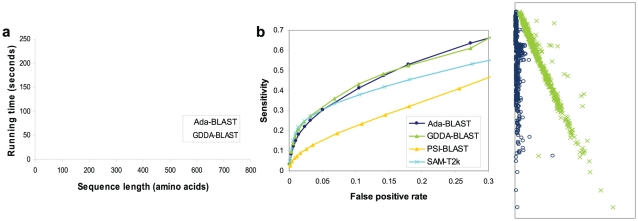
A Performance Comparison of GDDA-BLAST and Adaptive GDDA-BLAST. (a) Per-query running time of GDDA-BLAST and Adaptive GDDA-BLAST, when running 620 query sequences against 51 target sequences. The numbers in a box represent how much faster Adaptive GDDA-BLAST is than GDDA-BLAST. (b) Fold recognition performance of GDDA-BLAST, Adaptive GDDA-BLAST, PSI-BLAST and SAM-T2K on SABmark Twilight zone set is shown with ROC curves. 534 sequences of 61 SCOP fold groups from SABmark Twilight zone bechmark set. To calculate the sensitivity at different false positive rates, top-*k* sequences with the highest similarity to each 534 queries are considered as increasing k from 1.

To compare GDDA-BLAST and Adaptive GDDA-BLAST in terms of execution time, we ran both methods with the 602 query sequences and the 51 target sequences randomly chosen from the SABmark twilight zone set [26] and from the CDD database, respectively. Figure 2-a shows the per-query time for execution of alignment when a given query is run against the 51 PSSMs in the library. The lengths of the 602 query sequences range from 34 to 759 amino acids. Note that the running time of GDDA-BLAST linearly increases as a function of query sequence length. Conversely, Adaptive GDDA-BLAST shows much better scalability with respect to query size, because Adaptive GDDA-BLAST inserts a seed only at the positions where the seed is likely to be extended. Moreover, the performance gain is maximized when the two compared sequences are of low identity, because the number of the candidates for seed-inserting positions is limited. This makes Adaptive GDDA-BLAST an attractive alternative for alignment of highly divergent sequences. Overall, Adaptive GDDA-BLAST is on average 19.3 times (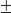
15.29 S.D.) faster than GDDA-BLAST, while it achieves more than 100 times speed-up in many occasions.

### Detection of Structural Homologues of High Sequence Divergence

To see if Adaptive GDDA-BLAST alignments could be used to encode informative alignment profiles for proteins, we took on the challenge of detecting structural homology in highly divergent protein sequences. For the test, we used 534 sequences from 61 fold groups in SABmark Twilight zone set. As employing 23,511 NBCI CDD PSSMs as a measuring PSSM set, each of 534 queries was encoded in an alignment profile. In this test, 60% coverage and 10% pairwise identity thresholds were used for filtering alignments. We used the Pearson's correleration coefficient to measure the similarity between two alignment profiles. We performed Receiver Operating Characteristic (ROC) curve analysis [4] to measure the performance of GDDA-BLAST and Adaptive GDDA-BLAST. The ROC curve shows the sensitivity of each method at different false positive rates. The sensitivity and false positive rates are calculated as follows: 
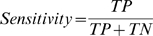
, 
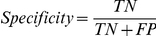
, 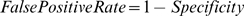, where *TP* is the number of true positives, *TN* is the number of true negatives and *FP* is the number of false positives. To calculate the sensitivity at different false positive rates for each query, we used the sequences with *k* highest Pearson's correleation coefficient for the query, while increasing *k* from 1 up to 40. As shown in Figure 2-b, the performance difference of Adaptive GDDA-BLAST, when compared with GDDA-BLAST, is negligible.

Figure S1-a shows that the percentage value of coverage has been effective to remove random alignments. We tested four cases including 40%, 60%, 85%, and no-coverage-threashold. It turned out that 40% and 60% coverage thresholds showed the best performance, followed by 85% and then by the no-coverage case. The 85% coverage was too conservative, while the no-coverage was too liberal. As a result, both failed to filter out random and noisy alighments effecively while informative alignments are kept. Figure S1-b shows that Pearson's correlation cofficient worked best among the distance metrics for the task at hand.

### Characterization of Transmemebrane Protein Structure

**Figure 3:**
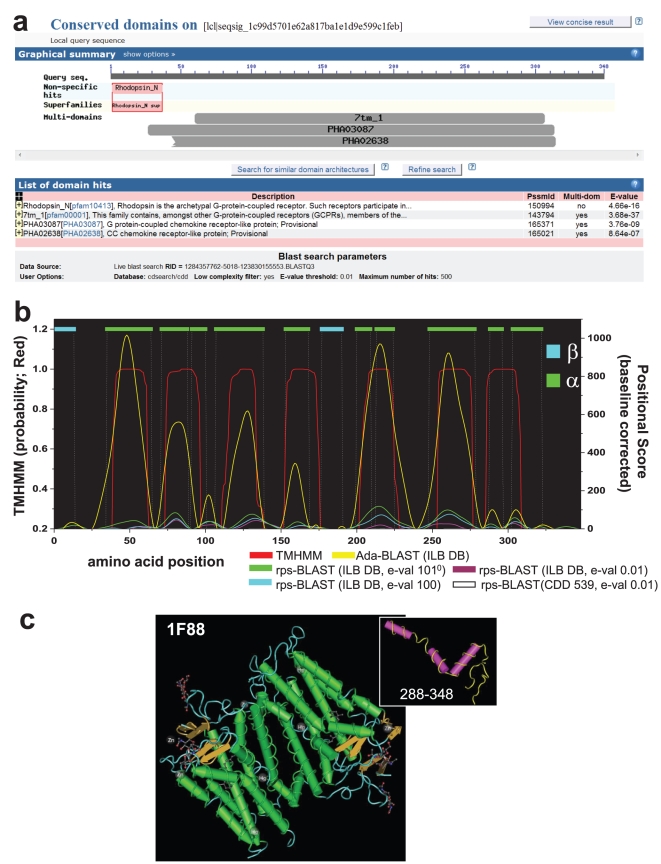
The Characterization of Membrane Spanning Regions. This graph shows the performance of the Hidden Markov Models (TMHMM), rps-BLAST and Adaptive GDDA-BLAST in determining the membrane-spanning domains in Bovine Rhodopsin as determined by X-ray Crystallography (Teal = Beta pleated sheets, Green = helices, loops not shown). This protein was analyzed with an expanded set of PSSMs representing a large variety of transmembrane domains (∼20K PSSMs). Compared with rps-BLAST, Adaptive GDDA-BLAST is more refined with respect to the annotation of alpha-helices. Moreover, this data demonstrates that less statistically valid alignments (e.g., e-value 0.01 vs. 10^10^) are still informative for detecting the domain boundaries and outperform lower thresholds. The full-length structure of Rhodopsin is shown (dimer) as well as an inset of the C-terminus that is composed of three small helices with the last one folding parallel with the membrane (it is not transmembrane itself).

We performed analyses on a structurally resolved (X-ray Crystallography) transmembrane protein called Bovine Rhodopsin (PDB: 1F88) in order to determine the information contained in a pure population of embedded alignments [27]. Figure 3-a depicts the result of rps-BLAST (e-value threshold 0.01) searching against NCBI CDD database. Notably, rps-BLAST returns overlapping alignments for 5 different PSSMs defined as Serpentine type 7TM domains as characterizing the domain architecture of 1F88. Based on structurally/functionally related PSSM libraries and the additional information below the accepted statistical thresholds, we define domain boundaries and secondary structural elements with higher resolution.

Our perfomance was evaluated against rps-BLAST and the TMHMM. TMHMM is a HMM-based method designed to predict transmembrane helices in proteins [28]. For this experiment, we considered a variety of executing conditions for rps-BLAST. To construct a PSSM library for transmembrane proteins, we first ran rps-BLAST against the CDD database, and consider, out of the alignments reported, only those PSSMs that we have previously annotated as transmembrane by keyword (CDD 539). Second, we also ran rps-BLAST against a database of PSSMs (n = 24,378) derived from expanding all of the sequences contained in the original 539 PSSMs, using PSI-BLAST (integral lipid-binding database, ILB DB) (see Methods). Finally, we ran rps-BLAST against these databases and slid the e-value threshold to less statistically significant levels (0.01, 100 or 10^10^).

It is resonable to consider that the amino-acids within transmembrane spanning helicies will be more conserved than the intervening loop residues. The support for this hypothesis is presented in Figure 3-b, wherein we report our results in comparison with the known structural elements of 1F88 obtained from the X-ray crystallography. The full-length structure of Rhodopsin is shown in the bottom right (dimer) as well as an inset of the C-terminus. The structural features are annotated with droplines (Cyan = Beta pleated sheets, Green = helices, and loops not shown). For each case, the transmembrane probability determined by TMHMM is shown on the left axis (red). The right axis represents a positional score for the Adaptive GDDA-BLAST and rps-BLAST conditions. The positional scores were quantified in the following manner: For each positive PSSM, the alignment boundaries were determined by the overlapping alignments obtained from either Adaptive GDDA-BLAST or rps-BLAST. These regions were extracted and realigned through the Smith-Waterman algorithm with BLOSUM62 and BLOSUM45 substitution matrix. Identical residues were scored as 2, and positive (i.e., non-identical but conserved) ones were scored as 1. This process was repeated for all positive PSSMs, and the results were summed for each amino acid in the protein. Unfortuantely, however, it was not sure which substitution matrix was optimal. Thus, we averaged the positional scores derived from BLOSUM62 and BLOSUM45. The positional results were normalized to the mean of zero by subtracting the average positional score from each point across the protein length. And then, each amino acid position underwent smoothing (Fourier-transform point = 8) and discontinous baselining using Origin Lab 7.5©. Baseline correction was performed by baselining the entire curve to every local minimum. These results demonstrate that: (1) use of the expanded ILB DB increases the signal-to-noise ratio by means of either rps-BLAST or Adaptive GDDA-BLAST, (2) Adaptive GDDA-BLAST has a ∼10-fold increase in signal compared to the largest results obtained from rps-BLAST (10^10^) and (3) the positional data correspond with the results obtained from TMHMM, even when tested with the expanded ILB DB under the highest statistical limit of rps-BLAST. In all cases, correlation exists between the curves obtained from the positional data and the known structural elements.

While both of TMHMM and Adaptive GDDA-BLAST do not accurately model the whole crystal structure, we observe interesting features. For example, several of the membrane-spanning helices are interrupted by loop regions that are not identified by TMHMM. Indeed, the C-terminus of 1F88 (aa 288–348) contains 3 small helices, the last of which is a bent-helix that is believed to be parallel to the membrane (Figure 3 *inset*). Both rps-BLAST and Adaptive GDDA-BLAST detect these smaller helices while Adaptive GDDA-BLAST has the highest signal. Another region of interest is contained between aa 91 and 111 which is a loop in the crystal structure, but is predicted to be a short helix by rps-BLAST and Adaptive GDDA-BLAST. We expect that this loop may be a bent-helix similar to other regions in the protein under native conditions.

### Characterization of Ankyrin-repeat Protein Structure

**Figure 4:**
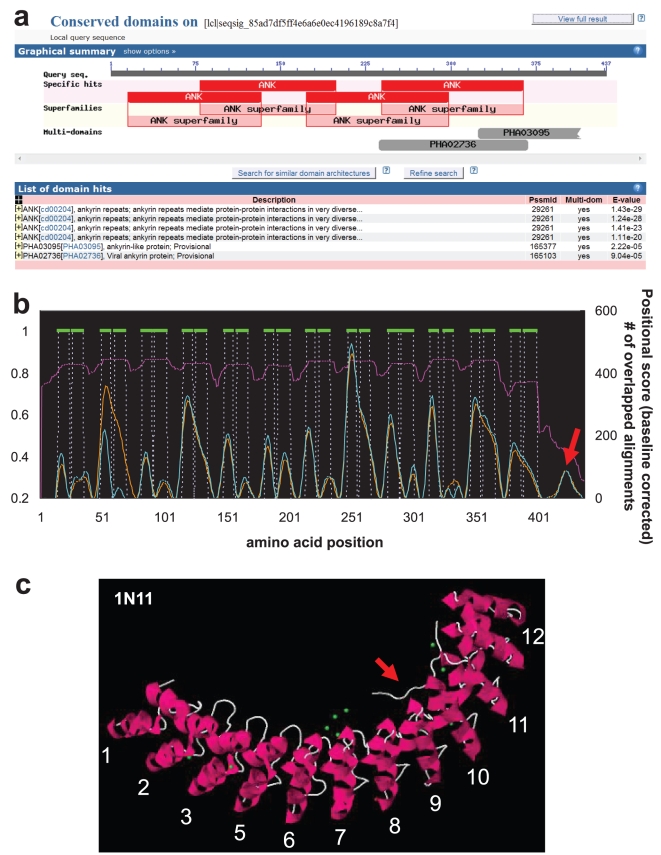
The Characterization of Ankyrin-repeat Protein Structure. This graph shows the performance of Adaptive GDDA-BLAST in determining the Ankyrin-repeat domains in Human Ankyrin-R as determined by X-ray Crystallography (Green = helices, loops not shown). This protein was analyzed with an expanded set of PSSMs representing Ankyrin-repeat domains (449 PSSMs). Adaptive GDDA-BLAST annotates 12 Ankyrin-repat domains as well as their alpha-helices. Compared to rps-BLAST, Adaptive GDDA-BLAST shows the structure of 1N11 in much refiner resolution (orange: Fourier-transform point = 7, cyan: Fourier-transform point = 8).

To further show the utility of Adaptive GDDA-BLAST, we did the same analysis on a structurally resolved Ankyrin-repeat protein called Human Ankyrin-R (PDB: 1N11) [29]. While TMHMM or similar methods do exist to characterize the structures of transmembrane proteins, there is no such method for Ankyrin-repeat proteins. Therefore, this protein is the perfect example of adding even more value to our method. 1N11 has 12 Ankyrin-repeats where each Ankyrin-repeat is composed with two alpha helices separated by loops (Figure 4-b). To see if we can characterize the structure of this Ankyrin-repeat protein, we first prepared 449 Ankyrin-repeat PSSMS that were generated with the domain sequences returned by a simple keyword search of “ankyrin repeat” against NCBI CDD database. We then ran 1N11 against the PSSMs by Adaptive GDDA-BLAST. Figure 4-a depicts the output of rps-BLAST (e-value threshold 0.01) which predict that 1N11 has 4 Ankyrin-repeat domains. In contrast, the overlapping alignments returned by Adaptive GDDA-BLAST depict the domain architecture of 1N11 with all 12 Ankyrin-repeat domains predicted (pink line in Figure 4-b). When we did the positional analysis as we did for 1F88, Adaptive GDDA-BLAST could see alpha helices in each Ankyrin-repeat even though the results are not yet perfect. It is interesting that we have a signal at the C-terminus of the protein which is long loop in the structure (red arrow in Figure 4-b). This long loop is associated with a small fragment of spectrin-binding domain [29]. Thus, it seems that the small signal appears due to the function of Ankyrin-repeat proteins mediating binding activities.

## Methods

### Definitions

Let the target sequence be *X* and the query sequence be *Y*. The length of sequence *X* is denoted as |*X*|. Assume that |*X*| and |*Y*| are *n* and *m*, respectively. A subsequence of 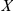 from the 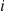-th residue to the 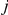-th residue is denoted by 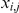 such that 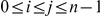. A subsequence of length one, such as 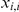, is simply represented as 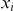. The concatenation of two sequences, *X* and *Y*, is represented as *X|Y*. Two subsequences that are aligned are represented within ( ). For example, 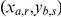 represents that 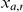 and 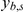 are aligned.

An embedded (chimera) sequence is generated by embedding either the N- or C-terminal portions of *X* as a “seed,” denoted by *S*, to every position of 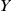. Usually *p%* of *X* (i.e., *k* residues of *X* where 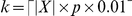) is used as a seed. For example, let 10% (*p* = 10) of a target be used as seed. If so, for a target of 100 amino acid long (|*X*| = 100), 10 residues (*k* = 10) of N- or C- terminal of the target are selected as a seed. Thus, N- and C-terminal seeds can be represented as 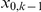 and 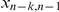, respectively. A chimera with a seed at the position 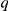 of 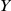 is 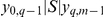, and represented as 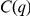. Table 1 shows an example of a chimera. To align the target sequence 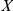 and the query sequence 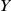, GDDA-BLAST generates 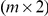 number of chimera sequences, inserting N- and C-terminal seeds from 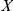 at each position of 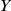 (Figure 1-ii-a). Each chimera is then aligned to 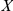, using rps-BLAST (Figure 1-iii). For each query, rps-BLAST is run independently, yielding a total of 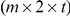 BLAST executions where *t* is the number of target sequences. Moreoever, the same procedure needs to be repeated for a total number of queries.

**Table 1:** Residues of a chimera sequence.

	y_0,q−1_				N-terminal seed (S)				y_q,m−1_			
Residue	y_0_	y_1_	…	y_q−1_	x_0_	x_1_	…	x_k−1_	y_q_	y_q+1_	…	y_m−1_
Chimera index	c_0_	c_1_	…	c_q−1_	c_q_	c_q+1_	…	c_q+k−1_	c_q+k+2_	c_q+k+3_	…	c_m+k−1_

This table shows an example of chimera sequence with an N-terminal seed of length 
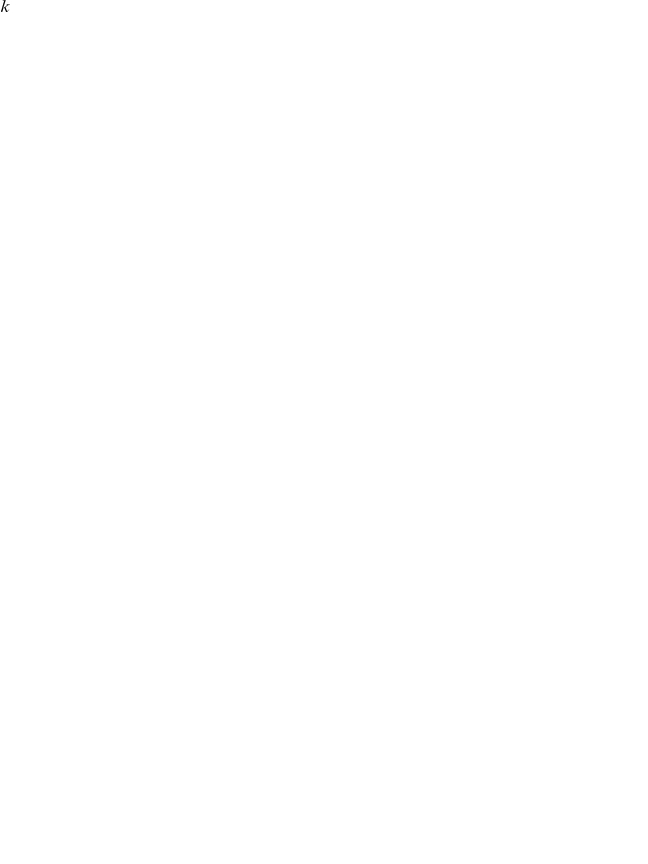
 inserted into the position 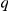 of the original query sequence Y. The length of the resulting chimera sequence is 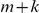, where 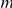 is the length of the original query sequence.]

Note that chimera sequences differ *only* by the position of the seed, implying that for two subsequent chimeras, a considerable part of the computation is recyclable. In the Adaptive GDDA-BLAST approach, we re-use the outputs from each step of rps-BLAST for efficiency purposes. For clarity, we hereunder define the outputs of each step. In the first step of rps-BLAST, we find hits between 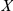 and 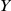. A hit of 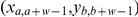, where *w* is a word size, is denoted as 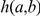. Upon extension of two neighboring hits without gaps between them, we obtain HSPs (High Scoring Sequence Pairs) in the second step. An HSP to align 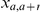 and 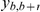 is denoted as 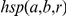. If an HSP has a score high enough to trigger gapped extension in the third stage, then an alignment is generated through extending the HSP with gaps in both directions from a residue pair in the highest scored region of the HSP. Note that the pair from which gapped extension is initiated is often referred to as a seed as well [24]. In order to avoid confusion, we denote this as a *GE starting pair* to distinguish it from the embedded seed of GDDA-BLAST.

### Observations of GDDA-BLAST

#### Observation 1. Seeding limits the search space

**Figure 5:**
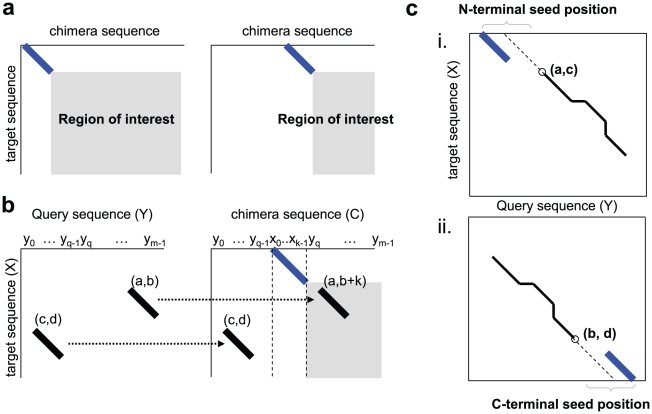
Adaptive GDDA-BLAST alignment details regarding seed embedding. (a) Limited region of interest with the seed embedding position. The diagonal line represents the alignment with the seed in different locations. The examples illustrate the region of interest of the N-terminal seeds. Similarly for the C-terminal seed, it is the upper-left corner of the seed. (b) The corresponding hits of a query and a chimera sequence. This example illustrates that the hits between the target sequence (X) and the query sequence (Y) can be reused for aligning a chimera sequence (C) against the target sequence (X). (c) The seed positions selected given a partial alignment. Ranges on the top and bottom represent the seed embedding positions of N-terminal and C-terminal seeds, respetively.

Since a seed provides an exact match, it is very likely that the GE starting pair exists in an HSP that includes the seed. Moreover, we are only interested in the alignments that include the seed, since the other alignments are locatable through the conventional methods using the original query sequence. This limits the search space of rps-BLAST. For example, when a seed is inserted at the position 0 of a query sequence, our search space will be the region in gray, as shown in Figure 5-a(left). Further, every time seed-inserting position is moved to the right, our search space is reduced, as is shown in Figure 5-a(right). Note that, in case of the chimeras with C-terminal seed, the search space is limited to the upper-left corner from the start position of the seed.

#### Observation 2. Chimeras share hits

Because chimeras are essentially the same sequence if the seeds are excluded, most of their hits are conserved. Therefore, the hits between X and Y are resuable in computation of the alignments of any chimera sequences. Consider a chimera, C(q). Let 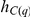 be a hit obtained, after the first rps-BLAST step between X and C(q). The relation between X-Y hits (i.e., 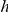) and X-C(q) hits (i.e., 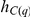) is defined, as follows:

*Lemma 1.*

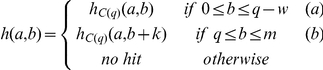
where 
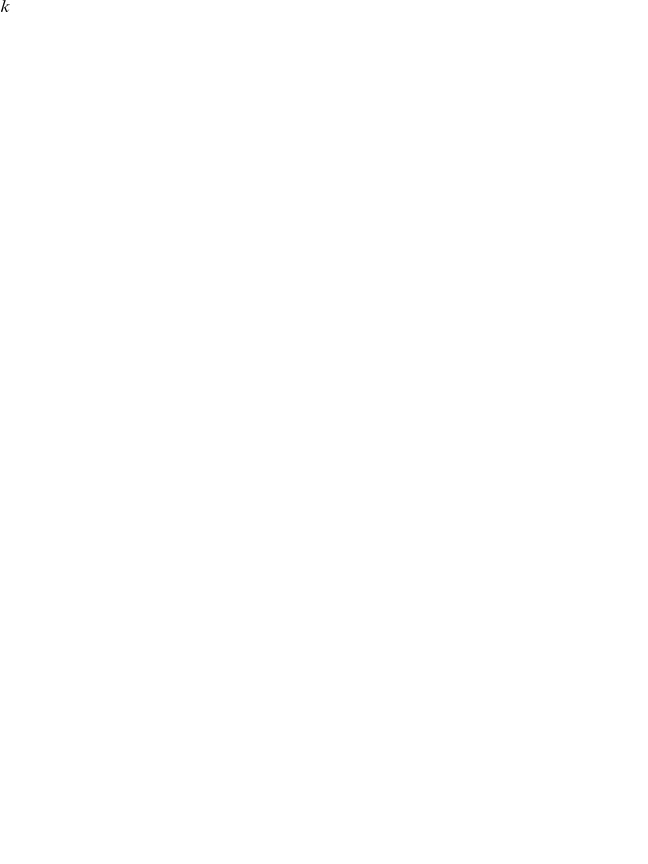
 is the seed length, 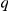 is the seed-inserting position and 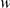 is the size of a hit.

Proof is omitted, since it is clearly shown in Figure 5-b. Note that we are *not* interested in the hits of lemma 1-(a) (Figure 5-b (c,d)), since they lie outside our interest, as demonstrated in Observation 1. Therefore, we only use the hits of lemma 1-(b) (Figure 5-b (a,b)) for alignment purposes.

#### Observation 3. Chimeras share HSPs after ungapped extension

rps-BLAST performs ungapped extension on neighboring hits, resulting in HSPs. Similar as in Observation 2, we define the relation between an HSP of X-Y (i.e., 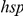) and that of X-C(q) (i.e., 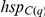), as follows:

*Lemma 2.*

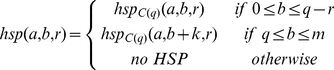
where 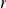 is the length of the HSP. The proof is straightforward from lemma 1.

#### Observation 4. Chimeras share alignment paths in gapped extension

**Figure 6:**
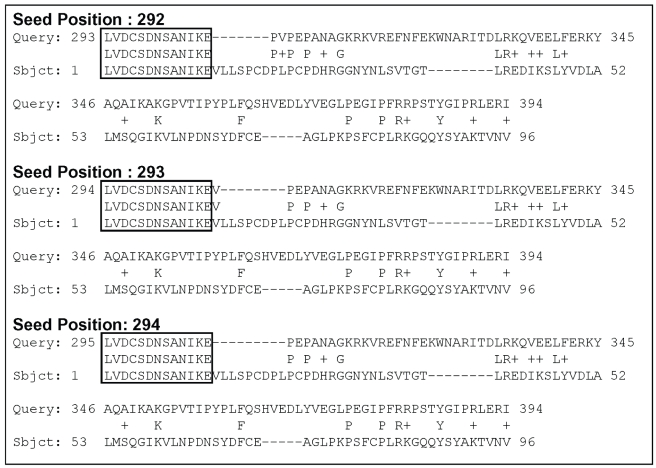
Example of embedded alignments. The seeded alignments for three consecutive chimera sequences. The query and the target sequences are general transcription factor II, i isoform from *Homo sapiens* (NP001509.2) and ML (MD
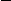
2
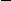
related lipid
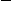
recognition) domain (cd00912), respectively.

Gapped extension in rps-BLAST starts at a *GE starting pair* that is a central residue pair in the highest scoring segment of any HSP whose score is sufficiently high. Different alignments can be generated, if the gapped extension is performed on different GE starting pairs and there is no guarantee that the same GE starting pair is selected for different chimera sequences. However, if a portion of a target sequence is conserved in a query sequence, then it is very likely that the conserved region is aligned for multiple neighboring chimera sequences. We exploit this property to speed up the alignment process (Figure 6).

#### Observation 5. Not every chimera produces a useful alignment

A seed provides artificial matches. It, however, is not extendable if there are insufficient neighbouring HSPs to connect to. Therefore, we can significantly reduce the computational complexity of the alignment process by inserting seeds only into a limited number of query positions that are likely to be extended. Hence, in Adaptive GDDA-BLAST, we align the query and the target sequence first, and then compute the seed-inserting positions from the alignment result, prior to aligning the chimera sequences.

### The Adaptive GDDA-BLAST Algorithm

**Figure 7:**
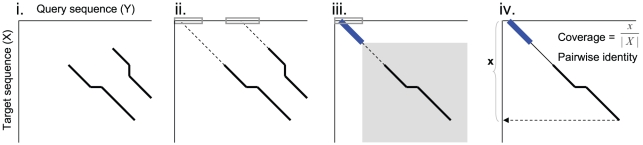
Four basic steps of Adaptive GDDA-BLAST. (i) Step 1: Find multiple non-overlapping local alignments. (ii) Step 2: Select seed embedding positions in query sequence. (iii) Step 3: Generate final alignments with seed. (iv) Step 4: Filter out non-significant alignments using coverage and pairwise identity of the alignment.

Adaptive GDDA-BLAST works through four basic steps, as shown in Figure 7. First, we find the conserved regions by generating non-overlapping local alignments between the query and the target sequence [30]. We call these *partial alignments*. Second, for each partial alignment from Step 1, seed-inserting positions are determined. Third, we produce *final alignments* including the seeds. Finally, we filter out the non-significant alignments, using quality parameters such as the %coverage and %identity of the alignment to the corresponding PSSM.

#### Step 1. Find multiple non-overlapping local alignments

Huang *et al.* [30] proposed an algorithm to find the multiple non-overlapping local alignments between two sequences. We have adopted this algorithm to generate partial alignments between the query and the target sequence. As a scoring matrix, we can use any given substitution matrix (e.g., BLOSUM62, BLOSUM45, PAM30, etc) or PSSM of a target sequence in our algorithm.

The local alignments are found, as follows: First, the hits between the query and the target sequence are found. A hit consists of three consecutive residues with a score larger than a threshold (i.e., *minimum word score*). For each hit, to generate an HSP, the ungapped extension is performed until the score drops below a threshold (*HSP drop-off score*). When a new HSP is constructed, the hits involved in the HSP are removed to prevent subsequent HSPs from extending over to them. This ensures that all local alignments produced later are not overlapped. We keep only the HSPs with scores larger than the threshold (*minimum HSP score*). Gapped extension is then performed for each HSP to generate partial alignments.

We keep only the partial alignments whose lengths are greater than the threshold (i.e., *minimum partial alignment length*). The minimum partial alignment length is proportionally determined to the length of the target sequence. Since a partial alignment is a locally most optimal alignment, it is not likely that a seed is extended further than the end position of the partial alignment. In the final step, a final alignment is chosen based on the the coverage of the alignment over the target sequence. This pre-filtered partial alignments are capable of removing the seed-inserting positions where a seed is not expendable to the final alignments with sufficient coverage.

#### Step 2. Select the seed embedding positions

In this step, the seed-inserting positions in the query sequence are selected, with the partial alignments obtained from Step 1. As discussed in Observation 1, a final alignment is generated by extending a seed from its end positions. Since a partial alignment is a locally optimal alignment, the extension of a seed can be converged to the partial alignment if a seed is inserted nearby and if the score of the path is high enough. An alignment is usually generated with HSPs connected with small numbers of gaps inbetween, because the relatively high penalty of gaps is used in the sequence alignment methods. In addition, the score of partial alignment on either side of a gap must be higher than the gap penalty [9]. The gapped extension usually commences from the seed to the partial alignment, since the score of the alignment with the seed is typically much higher than that of the partial alignment. For this reason, it is possible to simply compute the seed-inserting positions using the score of a seed and the distance from the seed to the partial alignment.

Given a seed *S* with the score *Score(S)*, the maximum gap *G(S)* (i.e., the distance from the seed to the partial alignment) is computed, as follows: 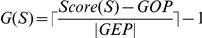, where *GOP* and *GEP* indicate a gap opening penalty and a gap extension penalty, respectively. Given a query sequence Y and a partial alignment (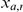, 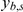), the query position *q* is subject to embedding of a seed with length *k*, as shown hereunder. (1) For N-terminal seed: 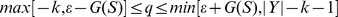, where 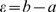; and (2) for C-terminal seed: 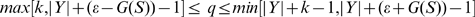, where 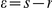. Note that the query embedding position *q* is computed in connection with the original query sequence positions. For example, if the N-terminal seed is inserted at the beginning of the query, then *q* is 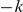 in order to preserve the original query sequence positions in the alignment. Recall that the region of interest starts immediately after the seed. Thus, in this way, we could preserve the original positions for the subsequent computations. The inserting positions for the C-terminal seeds are similarly represented. For C-terminal calculations, if 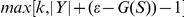 is larger than 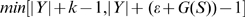, then no C-terminal seed is inserted. The idea of maximum gap has previously been described to connect the HSPs with gaps [9].

#### Step 3. Generate the final alignments with a seed

For each query position *q* identified in Step 2, we perform an alignment with the seed *S* inserted in its corresponding position to generate the final alignments. The final alignments are generated by running dynamic programming, starting at the end position of the seed, 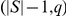, and by proceeding to 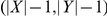.

Since we work with highly divergent sequences that produce low-identity alignments, it is reasonable to consider the following scenario: a longer alignment with a lower score can be biologically more meaningful than a shorter alignment with a higher score [11]. Motivated by this observation, during the alignment, we adjust an alignment score with respect to the length of the alignment as follows:
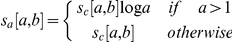
where 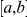 is a cell in the dynamic programming matrix, and 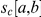 and 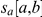 are the scores before and after adjustment, respectively. Note that *a* represents the alignment length at position 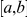 in the dynamic programming matrix. If we have the best score at 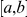, then we have the final alignment (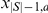, 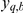).

#### Step 4. Filter out the non-significant alignments

Not all alignments produced from the previous step are informative. In this step, we prune out the insignificant alignments using the metrics, % *coverage* and *pairwise identity*. Given an alignment (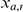, 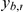), the percentage value of the alignment coverage to a target sequence is calculated, as follows:
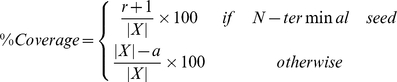
where *X* is a target sequence. The pairwise identity considered here is the identity of the alignment, excluding the matches in a seed: i.e., 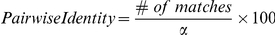, where 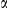 is the length of alignment excluding the seed. If the pairwise identity and the % coverage of a final alignment are greater than the thresholds (i.e., *minimum identity* and *minimum coverage)*, then the alignment is returned to the user.

### Experimental setup

Both GDDA-BLAST and Adaptive GDDA-BLAST were implemented in C, and compiled for both Linux and Windows environments. GDDA-BLAST utilizes rps-BLAST in NCBI BLAST 2.2.15 package to compute the alignments. In order to validate the approach, we tested both GDDA-BLAST and Adaptive GDDA-BLAST in terms of the execution time and the accuracy. The execution time experiment was conducted on a dedicated machine with 1.8GHz Intel Core
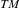
2 duo processor and 2GB memory running Windows Vista. The experiment on accuracy was performed on a server with eight Dual-core 2.4 GHz AMD Opteron processors and with a total of 32G memory running Linux. Note that, for the execution time experiment, we used a less-equipped dedicated machine instead of the server shared by others, in order to ensure the accuracy in the measurement.

### Generating function or structure-specific PSSM sets

To generate a PSSM set for a specific protein function or structure fold, we first collected the protein sequences, which were known to be related to the function or structure of our interest. For the PSSM set, we generated, using PSI-BLAST, PSSMs with the collected sequences or the sequences expanded from them. For expansion, each collected sequence is searched against NCBI NR database by PSI-BLAST (with the option of –e 10^−3^ and –h 10^−6^). Among the returned sequences, we filtered out any redundant sequences and the sequences whose pairwise identities to a query were more than 90%. For PSSM generation for those expanded sequences, PSI-BLAST (with the option of –h 10^−6^) was run again. All PSSMs used in this study will be provided upon request.

### Benchmark methods

For detection of structural homology, the ROC curves of GDDA-BLAST and Adaptive GDDA-BLAST were compared with those of PSI-BLAST and SAM-T2K. The settings used to run each method were: PSI-BLAST was run using NCBI NR database-added 534 query sequences, with the settings of a maximum number of 20 iterations (-j option), 0.0005 e-value threshold to include sequences for a profile construction at each iteration (-h option), and 1000 e-value threshold for returned alignment in the final iteration (-e option). For each query, all sequences that were aligned with the query out of 533 other sequences were sorted by their e-values. In case of SAM-T2K, *target2k* script in SAM 3.5 package was used for NCBI NR database search for each query, and multiple alignments of the returned sequences were generated. *w0.4* was used to generate an HMM model from the multiple sequence alignment. For each of the 534 queries, all 533 other sequences were scored based on the HMM model of the query by hmmscore with Smith-Waterman algorithm by default. Then, the 533 sequences were sorted out by their e-values.

## Discussion

In this study, we provide the supporting evidence for the following theories of ours: (1) that sequence embedding amplifies the low-identity alignments, and that these alignments are distinct from those derived through simple scaling of the e-value thresholds,; (2) that the low-identity alignments contain the information on protein structure/function; and (3) that PSSM libraries that are constructed from keyword searches of the CDD database, expanded using PSI-BLAST and then made into a specific database, can be used to classify proteins based on their structure/function. Several implications ensue from these findings.

How to biologically characterize the membrane-spanning proteins is a challenging problem. Likewise, the structural studies on this protein class are fraught with artifacts introduced by crystallization and/or lack of appropriate co-factors (e.g., lipids). Our results demonstrate that signals derived through Adaptive GDDA-BLAST could provide the structural information distinct from Markov Models of the transmembrane regions. Due to non-native conditions, it is still tantalizing to consider that these measurements may isolate potential discrepancies in protein structure.

The outstanding merit underlying the alignment profiles is centered on the successful construction of PSSMs representative of specific folds and/or activities. Indeed, the results from our present and companion studies [31] demonstrate that PSSMs libararies generated for a specific activity accurately identify homologous folds. Moreover, the activity-specific PSSM libraries also accurately identify homologous functions in proteins of diverse structure, as well as differentiate the activity within a specific fold. In this study, we generated transmembrane PSSMs curated by keyword searches; however, it is likely that additional refinement of this set (e.g., to remove non-transmembrane regions from PSSM) will lead to an increase in the signal/noise ratio, better annotation of transmembrane spanning domains, secondary structure prediction and more robust classification. We are actively pursuing our working hypothesis that this approach works for all types of protein domains.

Our results support the idea that statistical thresholds are often too stringent in domain detection algorithms. Through this study, it was found that additional information contained in alignments well below accepted statistical thresholds is also useable to identify domain boundaries and secondary structural elements. However, the finding was not applicable, when this same data were hierarchically clustered. Further analysis with a sufficiently large data set is required to identify and optimize the multiple variables that can identify highly divergent, yet informative alignments. Nevertheless, we propose that there is a wealth of information below statistical values, which is valuable to reserachers in annotating protein structure/function.

In conclusion, we propose that future works be aimed at (1) creating comprehensive and refined PSSM libraries and (2) exploring sequence embedding at the level of the PSSM (COBBLER [25]) and within the query (Adaptive GDDA-BLAST), will exponentially increase the functional annotation of all classes of proteins across taxa. Such advancement would have broad impacts on human health and disease, as well as basic science endeavours. Indeed, since Adaptive GDDA-BLAST performs in the “twilight zone” of sequence similarity, this approach may be harnessed to decode the most challenging protein datasets, and may also be scaled up to screen proteomes and the vast quantities of sequences being obtained from metagenomic studies. Outside of biological questions, the theories behind these algorithms are likely to have applications in numerous other fields that use pattern-based prediction algorithms.

## Supporting Information

Figure S1 Performance comparisons of different settings for GDDA-BLAST and Adaptive GDDA-BLAST. (a) Fold recognition performance of GDDA-BLAST when using different coverage threshold (with pairwise identity 10% threshold). It shows that %coverage is helpful to filter out noisy embedded alignments (no coverage threshold vs. 60% coverage thresholds). (b) Fold recognition performance of Adaptive GDDA-BLAST when using different distance metrics. Mutual information is estimated as described in [32]. 534 sequences of 61 SCOP fold groups from SABmark Twilight zone bechmark set. To calculate the sensitivity at different false positive rates, top-k sequences with the highest similarity to each 534 queries are considered as increasing k from 1.
(0.41 MB EPS)
